# Population genetic structure and gene flow of rare and endangered *Tetraena mongolica* Maxim. revealed by reduced representation sequencing

**DOI:** 10.1186/s12870-020-02594-y

**Published:** 2020-08-26

**Authors:** Jin Cheng, Huixia Kao, Shubin Dong

**Affiliations:** Beijing Advanced Innovation Center for Tree Breeding by Molecular Design, National Engineering Laboratory for Tree Breeding, College of Biological Sciences and Technology, Beijing Forestry University, Beijing, China

**Keywords:** Genetic diversity, Population genetic structure, Gene flow, Isolation by distance, *Tetraena mongolica* Maxim.

## Abstract

**Background:**

Studying population genetic structure and gene flow of plant populations and their influencing factors is of particular significance in the field of conservation biology, especially important for species such as rare and endangered plants. *Tetraena mongolica* Maxim. (TM), belongs to Zygophyllaceae family, a rare and endangered plant with narrow distribution. However, for the last decade, due to excessive logging, urban expansion, industrial and tourism development, habitat fragmentation and loss of natural habitats have become major threats to the population of endangered plants.

**Results:**

In this study, genetic diversity, population genetic structure and gene flow of TM populations were evaluated by reduced representation sequencing technology, and a total of more than 133.45 GB high-quality clean reads and 38,097 high-quality SNPs were generated. Analysis based on multiple methods, we found that the existing TM populations have moderate levels of genetic diversity, and very low genetic differentiation as well as high levels of gene flow between populations. Population structure and principal coordinates analysis showed that 8 TM populations can be divided into two groups. The Mantel test detected no significant correlation between geographical distances and genetic distance for the whole sampling. Moreover, the migration model indicated that the gene flow is more of a north to south migration pattern in history.

**Conclusions:**

This study demonstrates that the present genetic structure is mainly due to habitat fragmentation caused by urban sprawl, industrial development and coal mining. Our recommendation with respect to conservation management is that, all 8 populations should be preserved as a whole population, rather than just those in the core area of TM nature reserve. In particular, the populations near the edge of TM distribution in cities and industrial areas deserve our special protection.

## Background

Population genetic structure is the result of interaction between ecological and genetic processes. The study of genetic structure and gene flow play an important part in understanding the genetic characteristics and the dynamics of population, especially, those rare and endangered species with a limited geographical range. This is mainly because their populations are more vulnerable to damage, and gene flow is considered one of the most important determinants of the genetic structure of plant populations [1]. In addition to the influence of plant characteristics, historical events of the population, ecological factors, natural selection factors, interference of human activities, historical events and other factors will also lead to the formation of genetic structure in the population. Habitat fragmentation by human activities so seriously threatens the viability of plant populations that may result in shrinking population and even extinction [2]. The primary driver of population decline is that large and contiguous habitats were divided into isolated and smaller patches. Some research have shown that gene flow among populations or individuals even within areas that comprise largely natural habitat can be restricted by urban development [3], leading to habitat degradation and fragmentation [4].

Early studies relating to genetic diversity, population genetic structure and gene flow studies have largely relied on traditional molecular markers (Random Amplified Polymorphism DNA, Simple Sequence Repeats, Amplified Fragment Length Polymorphism, Restriction Fragment Length Polymorphism and so on). However, these markers have their own limitations in resolving the geographical differentiation of population, especially when using few genetic markers. In addition, developing traditional molecular markers not only takes time and effort, but also involves complicated steps, and most importantly they are costly. At present, whole genome sequencing is still pretty expensive, especially sequencing of hundreds samples. With the rapid development of high-throughput sequencing technology, so far, scientists have developed various reduced-representation genome sequencing by restriction enzyme digestion of genomic DNA [5–7], and the genotyping-by-sequencing (GBS) methods are increasingly applied to SGS and gene flow analysis [8–11]. GBS method can obtain a large number of SNPS, which allows us to resolve patterns of genetic diversity and spatial structure at very fine scale [12]. Compared to other molecular markers, GBS approaches have well suited to unravel genetic diversity, genotyping and genetic structure in nonmodel species without reference genome [13]. Employing large GBS datasets to biogeographic studies can vastly increase the accuracy and provide higher resolution relative to traditional molecular markers, especially in order to observe subtle changes in the genetic structure of the population.

*Tetraena mongolica* Maxim. (TM) which belongs to Zygophyllaceae family, is an endangered species. It is only distributed in the Alashan Ordos area of Inner Mongolia in china, and its habitats is defined as ecologically fragile areas due to the extremely low annual rainfall, TM which is dependent on its well-developed roots can thrive in these places and has gained considerate attention for being extremely resistant to drought and cold as well as windbreak and sand-fixation. However, owing to excessive logging (for firewood), overgrazing, urban expansion, industrial development, coal mining and gas exploration, together with habitat fragmentation, the number of TM have declined significantly in recent decades. The area of TM decreased by 225.2 km^2^ (from 629.5 km^2^ in 1990 to 404.3 km^2^ in 2010) declined by up to 35.8%, and the reduced area was mainly transformed into mining areas, sandy land, farmland and urban area [14]. Currently, TM has been classified as first-class protected plants in China. Previous studies have been mainly focusing on reproductive characteristics [15, 16], anatomy, vegetative cuttings [17], chemical components [18, 19], eco-physiological adaptation [20], landscape pattern [14], influence of mining [21], salt-tolerance and drought-tolerance genes [22, 23], genetic diversity analysis [24–26] and so on. In order to study the population structure and endangered mechanism of TM, it is vital to deeply delve into TM from the perspective of molecular biology and ecology. Therefore, studying TM population genetic structure and gene flow is of great importance for the preservation of this species and the ecosystem of this region. As a representative material for studying endangered species protection in desert ecosystems, TM has the following advantages: 1) limited distribution; 2) the biological community structure in TM distribution area is relatively simple, as TM is the dominant species there; 3) the potted history of urban construction in the TM area can give explicit knowledge that how human activities impose potential impacts on this region.

To understand the geographic patterns of genetic variation, population structure and gene flow of this plant will be crucial for their effective conservation. Reduced representation sequencing technology (SuperGBS method) is utilized in this study to evaluate patterns of genetic diversity, structure and gene flow of TM population across its entire geographic distribution range. Based on a genome-wide SNPs analysis, a novel understanding of genetic structure and gene flow of TM under urbanization, apparent hydrographical and geological barriers (Such as the Yellow River, ZhuoZi mountain) were revealed. Furthermore, this study also characterized biogeographic patterns of TM population and discovered several possible factors for its formation. Also, we assessed “isolation by distance” based on large-scale spatial genetic structure. According to the results, at the last, we put forward some constructive suggestions for better protection and follow-up management.

## Results

### SNPs discovery, genotype and population genetic diversity

One hundred twenty TM accessions were sequenced using Illumina Hiseq Xten, PE150 Platform, after the primary quality filtering step, a total of 981,376,270 reads, 133.45 Gb clean reads were generated, average of per accession were 1.11 Gb reads. According to the comparison results, the average sequencing depth of all samples was 104.17×, and the coverage ranged from 77.89 to 89.66%. With a minimal set of initial quality filters, we obtained 40,931 SNPs. After rigorously screening (standards: the missing rate < 0.2, MAF ≥ 0.01, DP > 4), 38,097 SNPs genotyping data were obtained, and several values of genetic diversity were calculated. With all 120 individuals, as a whole, it is observed that the Ne varied from 1.020 to 2 and average was 1.616, the levels of Ho and He ranged from 0.067 to 0.933 and 0.019 to 0.500 with an average of 0.446 and 0.348 respectively. Also, the PIC values varied from 0.019 to 0.375 with an average of 0.274 (Table 1), a total of 35,414 (92.95%) SNPs had a MAF greater than 0.2, a high degree of polymorphism (MAF > 0.30) was 18,800 and accounted for 49.34% of the dataset, the nucleotide diversity (π) varied between 0.019 and 0.502 with an average of 0.349. For each population, HN population had the highest He, Ne, PIC and π among all eight populations (average 0.341, 1.608, 0.269 and 0.354 respectively), whereas these four numbers was the lowest at SZS population (0.337, 1.599, 0.265 and 0.349 respectively). The above data indicate that TM had moderate level of genetic diversity on the whole. For different populations, HN population showed a higher genetic diversity than 7 other populations. According to the genetic diversity data and fine geographic scale analysis, HN population was middle ground of north-south intersection in the TG distribution areas. This is because north wind blows all year round and the Yellow River runs from south to north in the TM distribution area, and we speculate that HN population most likely accepted large amounts pollen from other populations or gene flow mediated by seeds. In addition, HN population is located at the national nature reserve of TM, and therefore, HN populations have higher levels of genetic diversity than other populations, this result is consistent with Ge et al. [25]. Conversely, SZS population lies at the southernmost tip of TM distribution area west of the Yellow River, the distribution area was adjacent to several big industrial parks in HuiNong district and backfill zone of open-pit coal mine, coal mining and industrial development have caused fragmentation and damage of SZS population local habitats. This result may suggest that the SZS population may have been experiencing a slowly reduction in genetic diversity as it is hugely affected by human activities.

**Table 1:** The number of individuals and genetic diversity statistics for each population, observed heterozygosity (Ho), expected heterozygosity (He), polymorphism information content (PIC), efficient allelic number (Ne), nucleotide diversity (π)

populations acronym	N	Ho	He	PIC	Ne	π
WJM	15	0.4588	0.3401	0.2675	1.6059	0.3522
TST	15	0.4586	0.3411	0.2683	1.6078	0.3531
QLS	15	0.4556	0.3374	0.2655	1.6006	0.3494
HN	15	0.4574	0.3419	0.2690	1.6086	0.3543
WD	15	0.4581	0.3410	0.2683	1.6070	0.3531
MHG	15	0.4547	0.3391	0.2668	1.6043	0.3512
SAS	15	0.4522	0.3372	0.2654	1.5996	0.3493
SDQZ	15	0.4579	0.3397	0.2673	1.6046	0.3520
all	120	0.4461	0.3482	0.2746	1.6169	0.3497

### Population structure and phylogenetic analysis

**Fig. 1:**
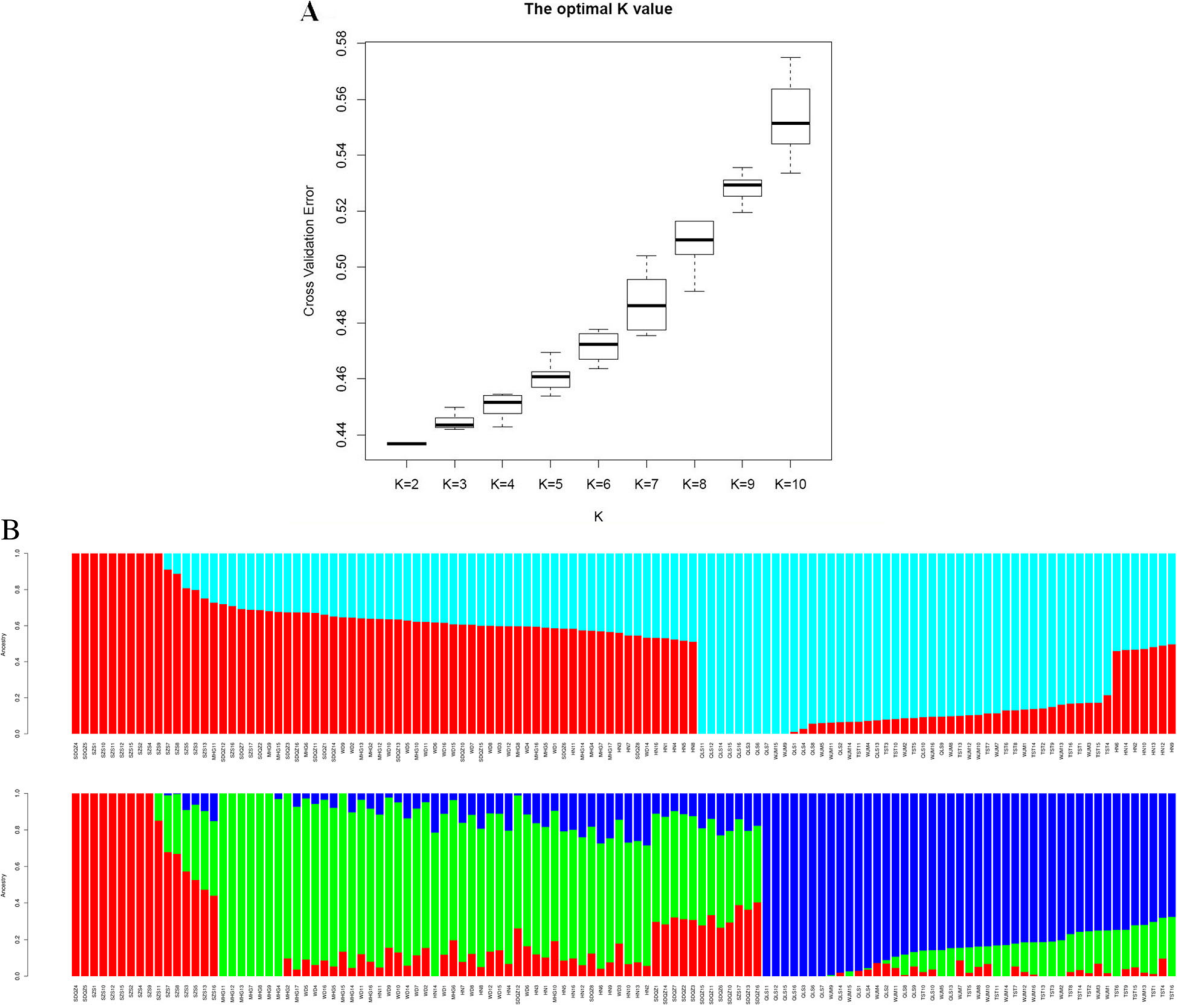
Population structure of 120 *Tetraena mongolica* individuals from 8 different geographical locations. **a** Cross-validation (CV) errors suggest that the 120 *Tetraena mongolica* individuals were divided into 2 genetic populations. **b** Population structure analysis with K = 2, K = 3. Each individual is represented by a vertical bar that is divided by K colored segments representing the likelihood of a membership to each cluster. The population codes are the same as those shown in Table 2

To understand the population genetic relationships among all TM individuals, ADMIXTURE (version 1.3.0) software was used to evaluate genetic structure, the K-value represent the number of clusters of all 120 TM individuals during the analysis, and the optimal K-value was selected according to CV. In this study, CV ranged from 0.436 to 0.575 (Fig. 1a). We mainly focused on two cases (K-value = 2 and K-value = 3), because they clearly showed the major structure of these 8 populations. When the CV was 0.436, the optimal K = 2, which indicates that 120 TM individuals from eight populations were divided into two groups with highest probability (Fig. 1b), one group was comprised of QLS, TST, WJM population’s all individuals and 7 individuals of HN population, and another group contained WD, MHG, SDQZ, SZS population’s all individuals and 8 individuals of HN population. Geographical locations were compared to analysis, the two groups were divided by Wuhai urban area as the boundary, HN population distributed in the nearby of the Hainan district, Wuhai city, its location was middle ground of north-south intersection in TM distribution area, it suggest that this population could be a pathway and bridge between the two groups, but on the other hand, urban expansion and human activities caused a certain degree of fragmentation of the TM population. At this scale, with an increase of values of K (K = 3), all 120 TM individuals from eight populations were split into three groups (Fig. 1b), first group was comprised of all individuals of QLS, TST, WJM remained grouped a single cluster (blue in Fig. 1b), and the second group contained four populations (WD, MHG, HN, 13 individuals of SDQZ). Furthermore, the third group mainly included SZS and 2 individuals of SDQZ. In terms of geographical distribution patterns, individuals in first group came from the north of Wuhai urban area, and four populations in second group distributed around the Wuhai urban area. Also, the third group comprised of two populations perched on the south of Wuhai urban area. Although there were also a large number of genotype admixtures, a certain genetic structure was found between different populations, we finally determined that K = 2 was the most suitable number of genetic cluster based on the experimental populations corresponded spatially with major geographical features.

**Fig. 2:**
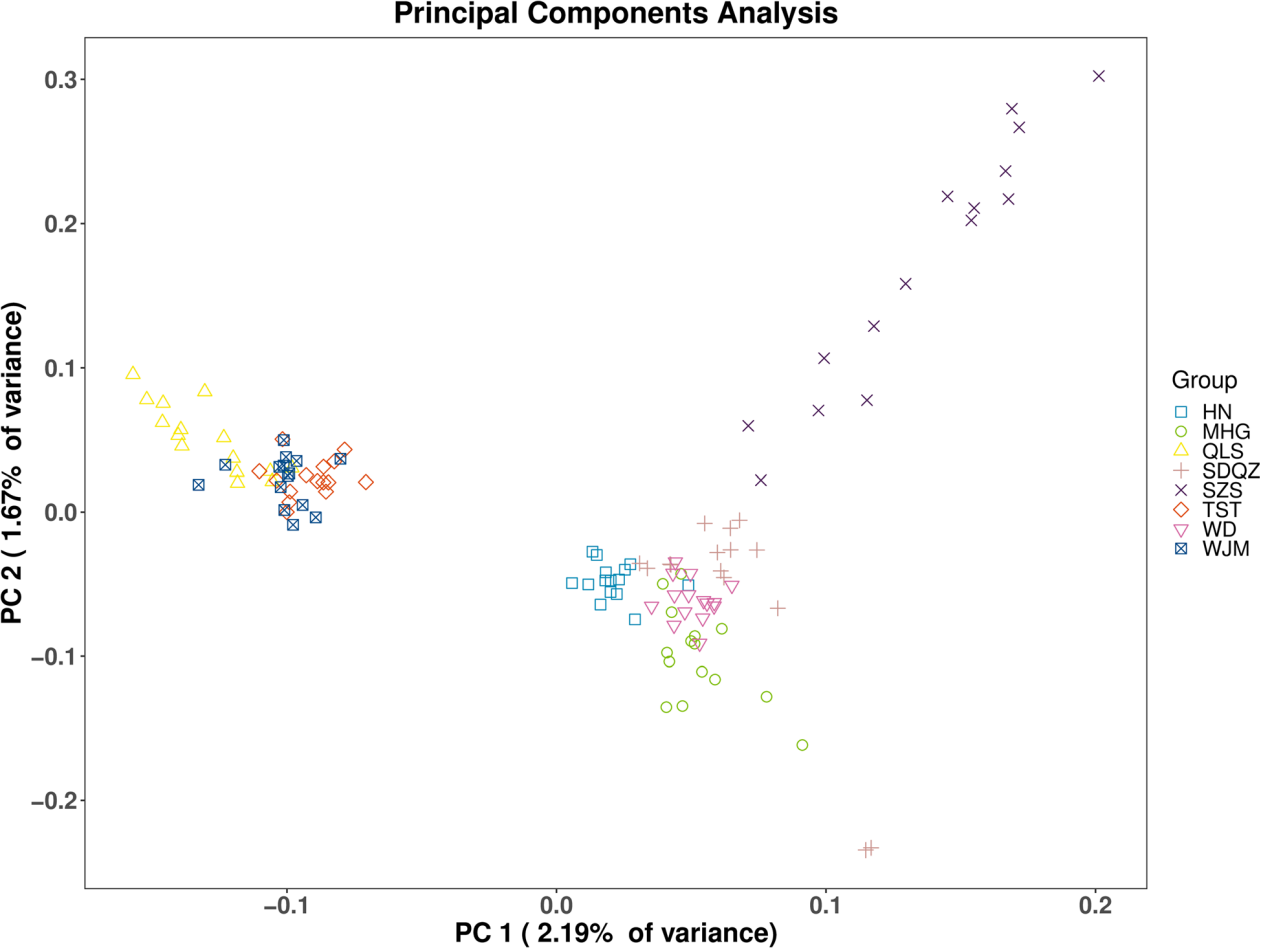
Clustering of *Tetraena mongolica* populations based on principal component analysis (PCA). Each point represents an individual colored according to the collection site

To complement ADMIXTURE analyses and to better understand genetic structure, our PCA analysis was based on obtaining SNPs among all 120 TM individuals. The reason why PCA was chosen is that it can detect fine scale genetic structure between populations, even there was extensive gene flow between these populations [27]. PCA analysis generally confirmed the pattern of genetic structure obtained with K = 2. Our results showed two principal components accounted for 2.19% (PC 1) and 1.67% (PC 2) of total variability (Fig. 2). All 120 TM individuals can be divided into two distinguished groups at PC1, one was comprised of QLS, TST, WJM population’s all individuals, and another one contained HN, WD, MHG, SDQZ and SZS population’s all individuals. In this way, the first group (QLS, TST, WJM) individuals showed low and negative values; on the contrary, another groups individuals displayed positive values. Under PC 2, 120 TM individuals roughly splited into three clusters: one comprising all individuals of QLS, TST, WJM population; the second is composed only of individuals of SZS population; third contained WD, MHG, SDQZ, HN individuals. The result of PCA analyses showed genetic clusters are similar to the result of ADMIXTURE analyses, illustrating a clear divergence between groups. Moreover, compared to other populations, high discretization of individuals of SZS implied this population was more heterogeneous and may be seriously disturbed. During survey research and sampling, we found that SZS was sandwiched between the Yellow River and the national highway and was close to the industrial park and backfill zone of open-pit coal mine in HuiNong district. Judging by this, it is a small population separated from early urban sprawl and industrial development, combined with our PCA data, this result further supported our hypothesis that SZS population was most disturbed by human activities.

**Fig. 3:**
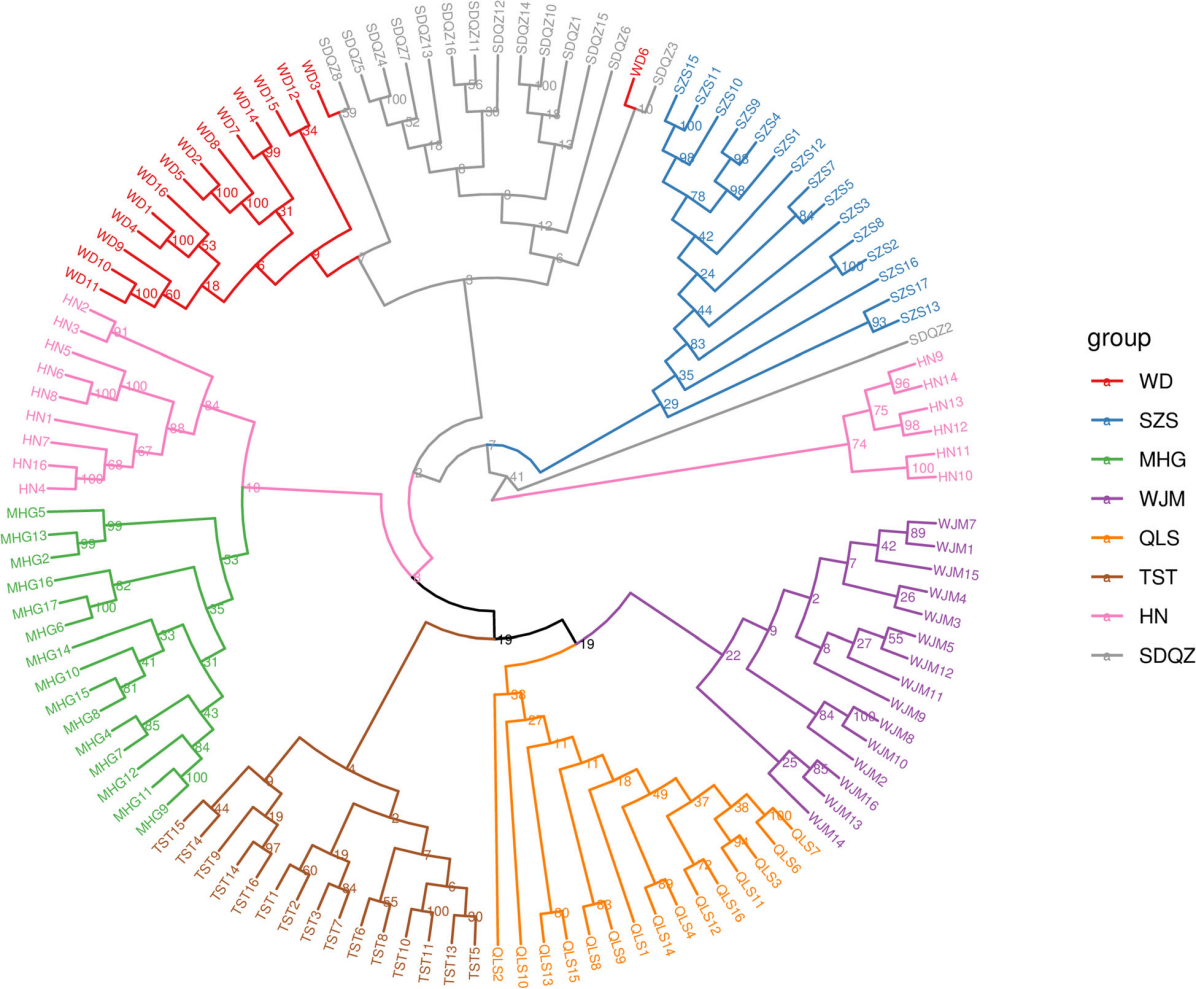
The neighbor-joining phylogenetic tree based on genetic distance matrix representing the grouping of 120 *Tetraena mongolica* individuals

To better visualise sample distribution and the relationships, a phylogenetic trees of 120 TM individuals were constructed (Fig. 3). Within the population, the individuals of each population get together except HN, but there are clear genetic boundaries between different populations. Fifteen individuals of HN population were divided into two parts and grouped into different clades: 9 individuals of HN population and MHG population got together, the rest 6 individuals of HN population clustered a clade alone, QLS, TST, WJM were close and grouped together into a clearly large cluster, WD and SDQZ clustered together (but a few individual position swapping, e. g. individual SDQZ8 and individual WD6). Fifteen individuals of SZS population clustered separately, SDQZ2 formed a branch alone, visibly, most genetic variation occurs primarily between populations of TM. In addition, HN population was assigned to different branches that suggested introgression exist.

Population structure, principal component and phylogenetic relationship indicated the existing 8 TM populations were divided into two groups with high genetic resolution. Zhi et al. [26] have evaluated the genetic structure in TM populations by 12 microsatellites SSRs, and their results showed no distinguishable genetic clusters were detected in populations. However, our study found the obvious genetic clusters and a sign that TM population is beginning to differentiation on the base of 38,097 SNPs genotyping data. We can also hypothesize that as the number of detected sites increased (38,097 SNPs VS 12 SSRs), more subtle genetic structures were discovered. Compared the PCA with fine geographic scale analysis, Wuhai urban area is the north-south boundary of TM population.

### Genetic differentiation and gene flow

Although the analysis of phylogenetic relationship and population structure showed that the TM populations were divided into two groups clearly, surprisingly, we detected very low levels of genetic differentiation and high levels of gene flow (Nm) between populations. And, genetic differentiation coefficient (Fst) ranged from 0.010 to 0.026 among 8 populations, and with an average of 0.020 within 120 TG individuals (Table 2). Wright [28] believed that if the Fst value of the populations was between 0 and 0.05, it will indicate that there is no genetic differentiation among populations, if Fst value is between 0.05 and 0.15, it is moderately differentiated, if Fst value is between 0.15 and 0.25, then it is highly differentiated. In this case, 8 TM populations without differentiation should be understood as a whole in our experimental. Our results on genetic differentiation are consistent with those of Ge et al. [24] and Zhi et al. [26]. Gene flow varied from 9.750 to 39.169 (average 12.25) between 8 populations in our study (Table 2). According to Wright [28], the Nm was divided into three grades: high (≥1.0), medium (0.250–0.99) and low (0.0–0.249) [29], when Nm > and 1 existed, there was certain gene flow between populations, our data suggested that there was high gene flow mediated by pollen or seeds between the 8 populations. In previous studies, by comparing the *atpB-rbcL* noncoding spacer region of the chloroplast DNA of TM, Ge et al. showed high levels of genetic differentiation (Fst of 0.38–0.90) and medium levels of gene flow (Nm of 0.04–0.71) between TM populations [26]. On the contrary, we found strong gene flow and almost no genetic differentiation between individuals or populations based on 38,097 SNPs. High intensity gene flow between 8 populations indicated Yellow River, Menggu Mountains, Zhuozi Mountains does not limit pollen movement and seed dispersal.

**Table 2:** Genetic differentiation coefficient and gene flow between different populations. The lower triangle is the interpopulation genetic differentiation coefficient (Fst), and the upper triangle is the interpopulation gene flow

populations	WD	SZS	MHG	WJM	QLS	TST	HN	SDQZ
WD	–	13.7478	19.8626	14.9107	12.6833	16.9795	21.7184	21.6031
SZS	0.01786	–	12.2876	9.75	9.0645	11.1655	12.9845	14.8284
MHG	0.01243	0.01994	–	13.1047	11.4926	15.2012	18.0650	17.0033
WJM	0.01649	0.025	0.01872	–	19.7021	39.1697	14.9107	13.8743
QLS	0.01933	0.02684	0.02129	0.01253	–	24.6009	13.2928	12.0230
TST	0.01451	0.0219	0.01618	0.006342	0.01006	–	18.8924	15.3750
HN	0.01138	0.01889	0.01365	0.01649	0.01846	0.01306	–	16.5624
SDQZ	0.01144	0.01658	0.01449	0.0177	0.02037	0.016	0.01487	–

The directions of the gene flow between different populations were also estimated. The result showed “north to south migration model” was the highest probability among the five speculative models (Table 3, based on the Bayes factor and the marginal likelihood). Although the numbers of gene flows shown there is a high level of gene flow between different populations, the north-to-south-migration model revealed the WJM population may have been an ancestral population that further spread southward coincided with prevailing winds.

**Table 3:** Log probability of the data given the model (marginal likelihood, based on the Bezier approximation score) and corresponding Bayes factors. The most probable model considers the north to south model

Model	Log (marginal likelihood)	Log (Bayes factor)	Model-probability
1: Adjacent	−497,724	−12,043.2	0
2: full migration	− 488,997	− 3315.64	0
3: north to south	− 485,681	0	1
4: south to north	−488,612	− 2930.91	0
5: Panmixia	−494,664	− 8983.39	0

### Correlation analysis between genetic distance and geographic distance

**Fig. 4:**
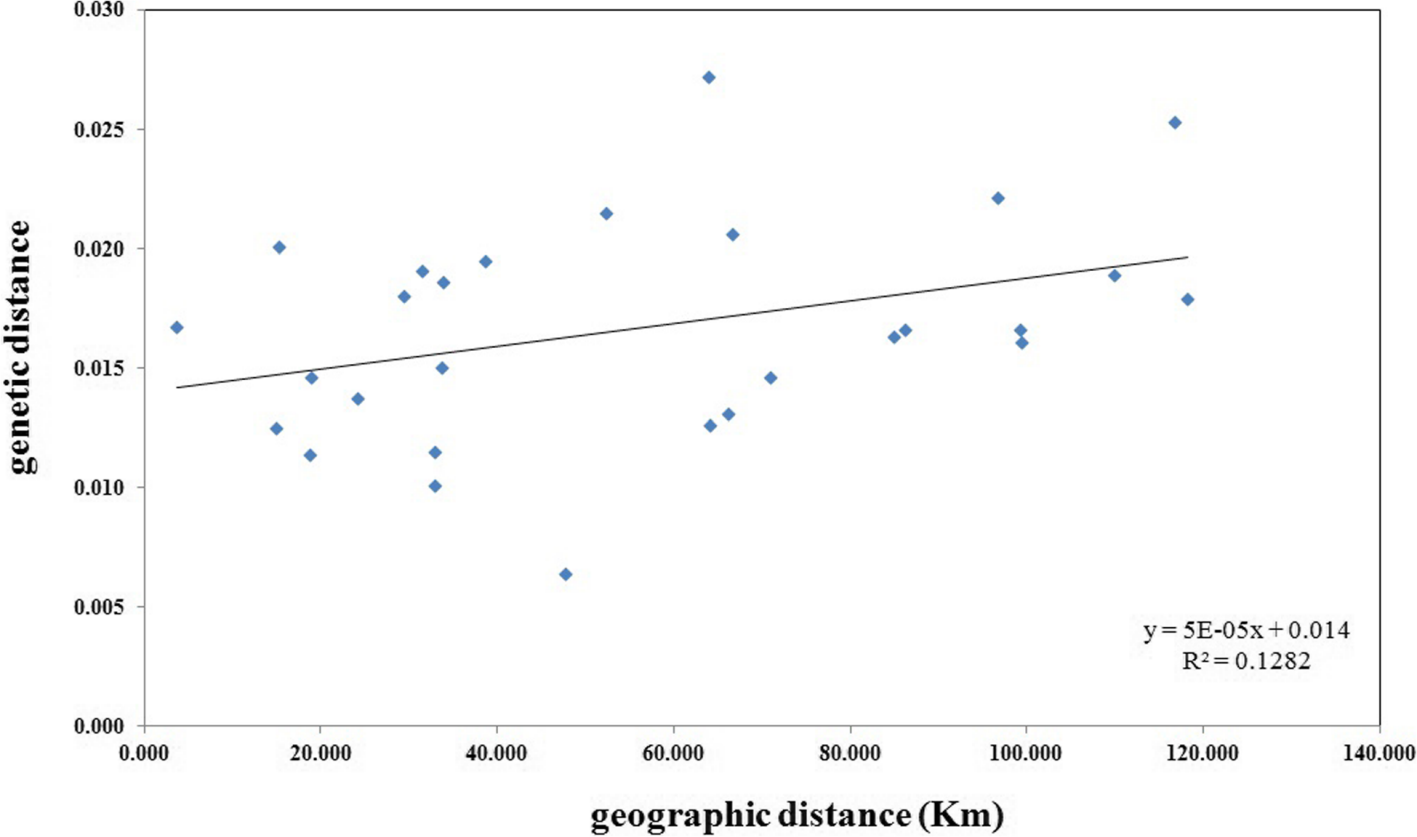
No significant correlations between geographic distance and genetic distance for 8 *Tetraena mongolica* populations was found through a Mantel test, *r* = 0.358, P (rxy-rand > = rxy-data) = 0.094. P (rxy-rand > = rxy-data) = probability of positive autocorrelation

To understand the effects of geographic distance on population, we computed the Mantel test and no significant correlation was detected between geographical distances and genetic distance for the 8 populations (*r* = 0.358, P (rxy-rand > = rxy-data) = 0.094) (Fig. 4). Although the distance between two most distant populations is more than 118 km, and some populations are separated by the Yellow River and Zhuozi mountain, “Isolation by distance (IBD)” model was not still found in our study. The result of the genetic distance of the fine geographic scale analysis suggests that geography distance is not the determining factor affecting the existing distribution pattern of TM. Even though habitat fragmentation can lead to isolation by distance [30], but most plants pollen flow may serve as a strong cohesive force [31], therefore, it is possible that high gene flow attenuate this isolation effect.

## Discussion

Spatial genetic structure is considered as a key factor in the short-term evolution of the population [32]. It is a result of the interaction between plant characteristics and ecological factors and it is likely to be influenced by human interference, historical events of the population, natural selection and other factors. For species with narrow distribution range, limited gene flow is the main influencing factor of small-scale spatial genetic structure. Species with limited gene flow are prone to the phenomenon of individual aggregation, thus forming the spatial genetic structure of population. TM is a monotypic genus of the Zygophyllaceae and a typical narrowly distributed endemic species, which lives in a very hostile environment. It it is the dominant species in its distribution area, the number and structure of TM population plays an important role in the overall biodiversity and ecological system in this region. However, genetic structure and the main influencing factors underlying relatedness of TM populations have not yet been addressed to date through fine-scale analyses of genomic data, previous studies conducted by Ge et al. [24, 25] and Zhi et al. [26] provide conclusions of the diversity and preliminary genetic structure analyses among existing populations using only a few molecular markers. In this research, we analyzed all of the TM population using reduced representation sequencing technology (the Super-GBS method), the average sequencing depth of the samples was 104.17×. Other relevant research have verified if the sequencing depth is more than 5×, the accuracy of genetic structure and gene flow analysis will be guaranteed [33, 34].

Through the analyses of nuclear genomic data we found some evidence of level of genetic diversity within this species. Several genetic diversity parameters from our study indicated whether 8 TM populations are considered as a whole, or populations separately, the finding only showed moderate levels of genetic diversity, which is largely different from the conclusion of Zhi et al. [26]. Geographical distribution is one of factors that affect the genetic diversity of plant species, species with wide range tend to have higher level of genetic diversity [35]. What’s more, our data show that the genetic diversity level of the population (HN) in the north-south intersection in the TM distribution area is higher than that of the marginal population (for example, SZS population). The HN population is an intermediate population for genetic exchange between the north and south populations, and it received more gene flow mediated pollen and seed. On the contrary, the SZS population was a marginal population that is located at the southern tip of TM distribution region. The low level diversity of SZS population indicated that it was seriously disturbed by human activities (habitat destruction caused by coal mining, industrial pollution, excessive logging). Also, individuals on the edge of the population are more likely to be killed by random events, resulting in loss of genes carried by these individuals, thereby reducing the genetic diversity of the population. Because other populations of TM are far away from the city, they experienced relatively little disturbance. According to ecological theory, in general, small populations of narrowly distributed species are expected to exhibit low levels of genetic variation, but high levels of genetic differentiation among populations [25]. Therefore, in addition to the core areas of nature reserves, small populations outside nature reserves need more protection.

Evidence of population genetic structure and gene flow presented here may provide a different insight and a previously undocumented subtle genetic structure for understanding current distribution pattern of TM populations. Population structure and PCA analysis showed that the existing 8 populations ware divided into two groups based on large-scale sequencing data, and the results were different from those of previous studies that have shown no apparent genetic structure [26]. In general, the Yellow River is a natural barrier for many species distributed in its basin, surprisingly, the two groups are not bounded by the Yellow River which is the second longest river in China. In contrast, in terms of geographic distribution, Wuhai city and its surrounding industrial areas are characterized as the boundary between this two groups. In addition, individuals of HN population were assigned to different groups and clusters in population structure and phylogenetic trees, it corresponds to the geographical distribution patterns, the HN population is located in the middle of the long and narrow distribution zone, and it revealed that the HN population is more genetic introgression and possesses more frequent communication with other populations.

The values of Fst and gene flow of among 8 TM population were measured, and the results showed no genetic differentiation and extremely high levels gene flow (Nm varied from 9.750 to 39.169) among different populations. Findings also indicated geological landforms such as Yellow River, Menggu Mountains, Zhuozi Mountains were not as barrier to gene flow, Thus, we speculated that it is this high level of gene flow that can sustain the current level of moderate diversity in this species. Additionally, a lot of admixtures were observed among in all populations in population structure. Some of the theories claim that admixture, reflecting allele sharing, can result from incomplete lineage sorting of historically contiguous populations [36]. It is also suggested all TM individuals in history are likely a continuously distributed large population connected by extremely large gene flow, in the case of such strong gene flow, how to understand the results of genetic structure and PCA analysis (The existing TM population were divided into two groups), apparently, the development of Wuhai city and the increasing number of industrial areas nearby have weakened the genetic exchange between the northern and southern populations to some extent, human activity interference was the most direct and major influence factors in recent decades [21], restricted gene flow is the main reason for the formation of genetic structure in population, the more restricted the gene flow between populations or between individuals, the more prominent the population genetic structure [37].

The direction of the gene flow was evaluated by migration model. According to our data, north to south migration model has the highest probability based on the marginal likelihood and the Bayes factor among the five speculative models (Table 3), this suggests that the northern population (WJM population, during the survey research, we found that WJM population had the largest distribution area and the largest number of individuals) may be ancestral populations and spread southward with prevailing winds in history. Pollen-mediated gene flow would be limited by the migration distance of pollination insects, plus gravity, the winds also cannot spread the seeds over long distances, and therefore, the main diffusion mode of gene flow is short distance step by step. Although all eight populations can be considered as one large group according to average 12.25 of gene flows, this is the result of long-term gene exchange after population expansion into stable habitats. In addition, gene flow in short distance can make the population with narrow distribution reach a dynamic balance by long-term evolution in the absence of interference, but once interference is present, it is likely to accelerate the generation of genetic structures. In addition, the Yellow River has made frequent floods, channel changes and running to lower flood plain in its history, may have had an effect on the fragmentation and diffusion of the population. On the other hand, the floods not only resulted in many habitats submerged, but at the same time carried the seeds across the river or helped the seeds spread to new areas. Therefore, compared to natural factors, the effects of urban sprawl and industrialization on adjacent migration of gene flow may be more dramatic.

In order to explore the spatial genetic structure of different source populations of species, Mantel test is often used to verify whether the species conform to the model of IBD, and to analyze the correlation between their genetic distance and spatial distance [38]. The results of Mantel test showed there is no correlation between geographical distances and genetic distance for all the 8 TM populations, this result was consistent with the high levels of gene flow that we detected. As an insect-pollinated plants with narrow distribution area, all 8 TM populations are in the similar or even same ecological environment, the genetic differentiation between populations caused by the ecological factor is not obvious, and the correlation between internal genetic distance and spatial distance is not significant as well. The biological characters of TM such as the high rate of ovule abortion and low seed setting percentage are considered as the main threats in the past studies [15, 39], additional threats including urban sprawl, industrial development and pollution, habitat fragmentation are expected to exacerbate the formation of genetic structures and the decline in biodiversity. In fact, it is expected that fragmentation and perturbation will reduce the effective size of population within patches and increase the genetic differentiation between the populations [40]. Urbanization can break up once-contiguous populations and leave the population patches scattered around the town [41]. Other relevant research showed that Wuhai is an industrial city with a short history of urbanization (about 60 years), and its industry is mainly coal-based energy industry [42], on the contrary, as a “living fossil” plant, TM has been existing in this area for thousands of years, other studies have shown that there is considerable overlap between the distribution of coal resources and TM in Wuhai area [21]. Therefore, the development of the coal mining industry and the process of urbanization are gradually splitting the TM habitat for a long time, habitat fragmentation induced by human disturbance has affected the genetic structure of TM population. Conservation biology agrees that larger populations survive longer, small populations that are separated from the whole can easily shrink or even extinction, which were all detected in this species.

## Conclusions

Combining genetic variation, gene flow, population genetic structure and IBD analysis, we can conclude that the existing genetic variation and spatial genetic structure of TM population were caused by rapid development of urbanization and industrialization. Although the TM population maintained a certain level of genetic diversity and a high level of gene flow, current populations has shown subtle spatial genetic structure, Wuhai city is in the middle of the north and south TM population, to some extent, the dense urban population can affect the routes of pollinators, insect-mediated gene flow over several generations that generated the current genetic structure, It is a signal that TM populations which are disturbed seriously by human activities beginning to started gradually forming genetic divergence. Compared with previous studies, our results of genetic diversity and population structure present a new perspective, and can provide valid information for TM management and conservation. To avoid genetic degradation and to maintain the TM population, we offer the following recommendations for protective measures: (1) Mining of opencast coal mines in TM distribution areas should be strictly forbidden, the establishment of new industrial zones in the TM distribution areas should be prohibited. (2) The 8 populations should be protected as a whole unit, as a bridge and link between the north and south populations. Also, the HN population should be continuously monitored and protected. In addition, because the populations have edge effects, those populations near urban and industrial areas also needs to be well-protected. (3) Collection of Germplasm resources, establishment of seed Banks and transplanting experiments are extremely necessary to expand TM population. Furthermore, as an insect-pollinated plants, and those pollinators should also be given adequate attention.

## Methods

### Sample collection and DNA extraction

In this study, we investigated roundly geographic distribution of TM before sampling, 144 individuals of TM were collected from all eight extant populations (The abbreviation of place name for populations were showed in Table 1). The sample collection was approved by the West ordos national nature reserve administration, Inner Mongolia Province, China. Eighteen individuals were chosen randomly in each population, in order to avoid clones sampling, each plant placed at a minimum distance of 50 m from each other were collected. The samples were carefully identified by Professor Zhixiang Zhang of Beijing Forestry University based on the descriptions in Flora of China, a voucher specimen was deposited in the Herbarium of Plant Biology Department, Beijing Forestry University with an accession number BJFU-TM005. Healthy leaves from 144 individuals were collected and were dried directly with silica gel in the wild, then taken back to the lab and frozen in − 80 °C until DNA extraction.

DNA extraction of each individual was carried out by DNAsecure Plant Kit (Tiangen Biotech, Beijing, CHN) following the manufacturer’s introduction. After DNA quality and concentration were tested by Agarose Gel and NanoDrop, we selected 15 samples with the highest DNA quality from each population as our experimental samples.

### Preparation of libraries and sequencing

we adopted modified GBS methods (SuperGBS, following Qi et al. [43]) to construct libraries. Briefly, extracted DNA from each individual was digested with both PstI-HF and MspI (New England Biolabs, NEB) for 2 h at 37 °C and then 2 h incubation at 75 °C. The barcoded adapters and common adapter were respectively ligated on the PstI cut site and the MspI cut site of all samples by T4 DNA ligase (NEB), ligation was running at 22 °C for 2 h. Fragments smaller than 300 bp were removed using recovery system of improved magnetic bead. The recovered fragment was amplified by PCR using high-fidelity enzymes, before libraries were sequenced, the concentration of PCR product was tested by Qubit2.0 and it should be greater than 5 ng/ul. Final libraries were sequenced using Illumina Hiseq Xten, PE150 Platform.

### Cleaning of the raw reads and SNP/indel calling

To ensure the accuracy of analysis, ‘FastQC’ (v. 0.11.4) [44] was used to test the raw read quality. The adaptor and barcode of raw reads sequence were cleaned by the process_radtags program in the STACKS (v2.1) [45] software package. The restriction site sequence, base quality < 20; and the last 5 bp of raw reads that were more likely to contain errors was removed using the FASTX_trimmer program package (main parameter -f-l) in the FASTX toolkit (v0.0.1 4) (http://hannonlab.cshl.edu/fastx_toolkit/). Through the steps above, clean reads were obtained. Because the public databases does not contain full genome information for TM, and even there is also no the genome data of relative species of zygophyllaceae family to reference. Under these circumstances, de novo generation of a GBS reference was constructed following [43]. Clean Reads were aligned against the GBS reference genome using bowtie2 (v2.3.4.1) software (main parameter -maxins 300 -no-discordant -no-mixed) [46], and then genotyping was performed applying Unified program in the GATK (v3.8–1) software (Main parameters -dcov 1000-GLM BOTH) [47] to predict SNP&INDEL sites in samples. The predicted results were screened using the SelectVariants program of the GATK software (Key parameters: - restricelesto biallelic-select “QD > 10.0”). In order to reduce the error rate of detected SNP&INDEL, we used the software vcftools (v0.1.13) (main parameter -maf 0.01 -minDP 4 -max-missing 0.8) to filter the obtained SNP typing results. The filtering conditions were as follows: (1) reads support (total depth, DP) is no less than 4; (2) eliminate the sites with minor allele frequency (MAF) less than 0.01; (3) eliminate the sites where the missing rate of SNP typing is higher than 20%.

### Genetic diversity analysis

Several population genetics general statistics to describe genetic variation were estimated by R package genepop (Version1.0.5) as described [48]. Mean number of alleles (Na), effective number of alleles (Ne), observed heterozygosities (Ho), expected heterozygosities (He), polymorphism information content (PIC) can be calculated according to the formula [49]. Vcftools software (Version 0.1.14) [50] was used to calculate the nucleotide diversity (π).

### Population structure, phylogenetic trees and principal components analysis

For further elucidation of the genetic structure of the eight TM populations, we used the PLINK (version 1.9) software (main parameter -indep-pairwise 50 10 0.6, 0.6 is r^2^ threshold) to filter the obtained SNP, the SNP without close linkage was selected, and then we used the ADMIXTURE (version 1.3.0) software [51] to estimate the genetic structure, the pre-defined genetic clusters (K) was set from 2 to 10, and 10 different models were selected for repeated analysis for 10 times. The optimal number of K was determined according to Cross Validation Error (CV) to in ADMIXTURE analysis.

To better understand and describe the genetic structure, the obtained SNP markers were analyzed by principal components analysis with plink2 software (version: 2.0) [52], and the two eigenvectors with the greatest influence were obtained.

Phylogenetic trees are tree branch structures representing near or far relationships between individuals, for further elucidation of genetic distance between samples and clusters. As the first step, each individual SNP marker was end to end, and if the corresponding locus was missing, the - was used instead. Neighbor-joining method was used to build phylogenetic tree of all 120 individuals, Treebest software (v1.9.2) [53] was used to calculate the distance matrix, the reliability of phylogenetic tree was tested by bootstrap method with 1000 replicates.

### Genetic differentiation and gene flow pattern inference

Genepop program (Version1.0.5) in R soft was used to analyze Fst of each SNP locus in all individuals. We also analyzed the gene flow level among eight populations and the parameter Nm was used evaluated using formula Nm = (1-Fst)/4Fst. The directions of the gene flow between different populations were estimated using MIGRATE (https://peterbeerli.com/migrate-html5/index.html) according to Beerli & Palczewski [54] and Blanco-Bercial & Bucklin [55]. The estimated models of gene flow directions were chosen based on the hydrography and prevailing wind of the region. We assumed five possible models: (i) adjacent, migration occur between adjacent populations only; (ii) full migration of north 3 populations (WJM, TST, QLS), full migration of south 4 populations (SZS, SDQZ, MHG, WD), Wuhai city to the north and south as a boundary, the intermediate HN population is a bridge; (iii) north to south, the direction of the prevailing wind in this area (pollen and seeds are dispersed by the wind); (iv) south to north, the direction of the Yellow River in this area (seeds may be dispersed along Yellow River); (v) panmixia, all samples were considered as a whole population (the movement of pollinators is random). For each model, marginal likelihood and the Bayes factor was used estimated possible direction of migration and the specific probability, the number of recorded steps in chain was set to 2000,000.

### Correlation analysis between genetic distance and geographic distance

In order to understand whether there is a correlation between genetic distribution and spatial location of TG from different populations, Mantel test is performed using GenAlEx v6.5 [56] with 10,000 permutations.

## Data Availability

The plant materials were collected from natural population in geographic distribution of TM. The raw fastq reads files can be accessed on NCBI Sequence Read Archive (SRA), accession nos. BioProject: PRJNA601311; BioSample: SAMN13870224.

## Abbreviations

SNP: Single nucleotide polymorphism
Ho: Observed heterozygosity
Na: Mean number of alleles
Ne: Effective number of alleles
He: Expected heterozygosities
PCA: Principal component analyses
PIC: Polymorphism information content
MAF: Minor allele frequency
GBS: Genotyping-by-sequencing
